# Early season estimate of influenza vaccination effectiveness against influenza hospitalisation in children, Hong Kong, winter influenza season 2018/19

**DOI:** 10.2807/1560-7917.ES.2019.24.5.1900056

**Published:** 2019-01-31

**Authors:** Susan S Chiu, Mike YW Kwan, Shuo Feng, Eunice LY Chan, Huiying Chua, Joshua SC Wong, JS Malik Peiris, Benjamin J Cowling

**Affiliations:** 1 Department of Paediatrics and Adolescent Medicine, Queen Mary Hospital and Li Ka Shing Faculty of Medicine, The University of Hong Kong, Hong Kong Special Administrative Region, China; 2 Authors contributed equally to the work and share first authorship; 3 Department of Paediatrics and Adolescent Medicine, Princess Margaret Hospital, Hong Kong Special Administrative Region, China; 4 WHO Collaborating Centre for Infectious Disease Epidemiology and Control, School of Public Health, Li Ka Shing Faculty of Medicine, the University of Hong Kong, Hong Kong Special Administrative Region, China

**Keywords:** influenza, H1N1, vaccine effectiveness, public health, Hong Kong, inactivated influenza vaccination, children, hospital, winter

## Abstract

The winter 2018/19 influenza season in Hong Kong has been predominated by influenza A(H1N1)pdm09 as at January 2019. We enrolled 2,016 children in three public hospitals in Hong Kong between 2 September 2018 and 11 January 2019. Using the test-negative approach, we estimated high early season effectiveness of inactivated influenza vaccine against influenza A or B of 90% (95% confidence interval (CI): 80–95%) and 92% (95% CI: 82–96%) against influenza A(H1N1)pdm09.

In Hong Kong, the 2018/19 influenza season started in October 2018, with influenza infections reaching epidemic levels in the final week of 2018, and influenza A(H1N1)pdm09 viruses circulating predominantly [1,2]. Previous influenza A(H1N1)pdm09 epidemics occurred in 2012/13, 2013/14 and 2015/16 [3,4]. Here we continued an ongoing study [3-5] using the test-negative design [6,7] to estimate influenza vaccine effectiveness (VE) in hospitalised children in Hong Kong in the early part of the 2018/19 winter influenza season.

## Study participants

In Hong Kong, ca 90% of inpatients are treated in public hospitals. We conducted a test-negative study among children aged 6 months–17 years in three public hospitals; Queen Mary Hospital, Princess Margaret Hospital and Yan Chai Hospital, which together have a catchment area covering ca 17% of all children living in Hong Kong. We enrolled children who were admitted with a febrile acute respiratory illness (ARI) to the general paediatric wards in these three hospitals. Febrile ARI was defined as fever measured ≥ 38 °C plus any respiratory symptom such as cough, sore throat or runny nose [3-5]. Healthcare staff collected nasopharyngeal aspirates from all enrolled children and tested for influenza viruses by reverse transcriptase PCR.

Influenza vaccination history of each patient was obtained by interviewing parents or legal guardians using a standardised questionnaire. Detailed vaccination history was further clarified by reviewing vaccination cards, contacting private clinics and/or checking electronic medical records. Children vaccinated within 6 months before hospital admission with an appropriate schedule according to the Advisory Committee on Immunization Practices [8], with the last appropriate dose at least 14 days before the hospital admission were categorised as vaccinated. Those who received influenza vaccination within 14 days of the hospitalisation were excluded from the analysis. Conditional logistic regression models were used to estimate VE, accounting for confounding by adjusting for age and age-squared and matching by calendar week. VE was estimated via 1 minus the adjusted odds ratio of vaccination [9]. All statistical analyses were performed in R version 3.5.0 (R Foundation for Statistical Computing, Vienna, Austria).

The study protocol was approved by the Institutional Review Board of the University of Hong Kong/Hospital Authority of Hong Kong West Cluster and the Kowloon West Cluster Research Ethics Committee. Verbal consent was obtained from parents or legal guardians of participants.

## Influenza vaccine effectiveness estimate

In order to prevent influenza infections and severe outcomes inactivated influenza vaccines (northern hemisphere formulation) are available, almost all of which are quadrivalent, and more than 1 million doses have been administered in Hong Kong during the 2018/19 season [10] in the local population of 7.4 million. Priority groups for vaccination include older adults, adults with chronic medical conditions, pregnant women, healthcare workers and children up to the age of 12 years.

From 2 September 2018 to 11 January 2019, a total of 2,016 children admitted to the three study hospitals were eligible to be included in the study (Figure). Of these, 344 (17.1%) tested positive for influenza A or B. Among the test-positives, 85% (292/344) were positive for influenza A(H1N1)pdm09 and 13% (45/344) were infected by influenza A(H3N2) viruses (Figure, Table). Among the influenza-positive children only 2.9% (10/344) were vaccinated, compared with 10.2% (170/1,672) among influenza-negative controls (p value < 0.001). Among the 180 vaccinated children, most (n = 160: 88.9%) received the quadrivalent inactivated influenza vaccine, compared with 10 (5.6%) who reported receipt of a trivalent inactivated influenza vaccine and 10 (5.6%) unknown. Influenza VE was 90% (95% confidence interval (CI): 80–95) against influenza A or B, and 92% (95% CI: 82–96) against influenza A(H1N1)pdm09.

**Figure:**
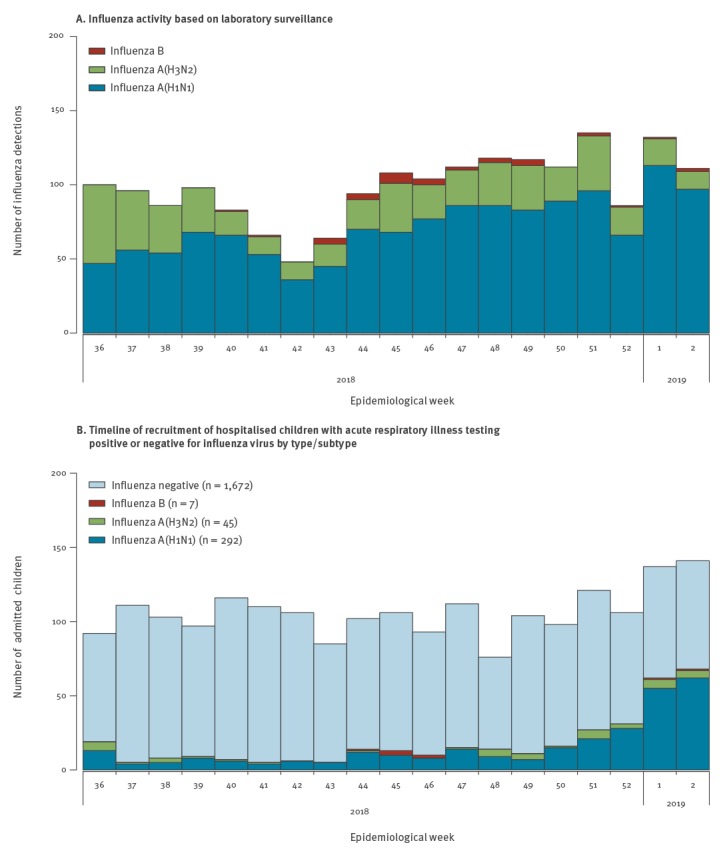
(A) Influenza activity based on laboratory surveillance^a^ (B) timeline of recruitment of hospitalised children with acute respiratory illness testing positive or negative for influenza virus by type/subtype, Hong Kong, 2 September 2018–11 January 2019 (n = 2,016)

^a^ Reported by the Public Health Laboratory Services.

**Table:** Comparison of cases testing positive for any influenza virus and test-negative controls, Hong Kong, September–December 2018 (n = 2,016)

Characteristics	Influenza-positive N = 344		Influenza-negative N = 1,672		P value
	n	%	n	%	
**Age group**					
6 months–2 years	130	37.8	846	50.6	< 0.001^a^
3–5 years	141	41.0	468	28.0	
6–17 years	73	21.2	358	21.4	
**Sex**					
Male	159	46.2	949	56.8	
Female	185	53.8	723	43.2	< 0.001^a^
**Receipt of influenza vaccination^b^**					
**Overall**	10	2.9	170	10.2	< 0.001^c^
**By age group**					
6 months–2 years	1	0.8	47	5.6	
3–5 years	5	3.5	68	14.5	
6–17 years	4	5.5	55	15.4	
**By sex**					
Male	0	0.0	94	9.9	
Female	10	5.4	76	10.5	

^a^ P values estimated by chi-squared tests.

^b^ Receipt of influenza vaccination defined as receipt of a quadrivalent or trivalent inactivated influenza vaccine with an age-appropriate schedule within 6 months prior to admission to one of the three study hospitals.

^C^ P value estimated by Fishers exact test.

To confirm that there was no evidence of a change in VE concurrent with the rising phase of the winter epidemic, we conducted a sensitivity analysis. We restricted the analysis to the 783 children admitted between 25 November 2018 and 11 January 2019. In this subgroup we estimated a VE of 87% (95% CI: 75–94) against influenza A or B overall and 92% (95% CI: 81–96) against influenza A(H1N1)pdm09.

## Discussion

In this study, we found high VE for influenza A(H1N1)pdm09. Our results suggest that the influenza vaccine was effective in reducing hospitalisations due to influenza A(H1N1)pdm09 in children in the early phase of the 2018/19 winter influenza season; highlighting the importance of promoting influenza vaccination in Hong Kong. Vaccination coverage was only 10% in the test-negative controls. A pilot school-based influenza vaccination programme was launched in October 2018 and expansion of this scheme could increase vaccination coverage in school-age children. Further strategies may be needed to improve vaccination coverage in children below 6 years of age, particularly in the age range of 6 months–2 years.

This is one of the first in-season estimates of influenza VE for the 2018/19 influenza season. Our findings were consistent with previous estimates against influenza A(H1N1)pdm09 in children in Hong Kong from 2012/13 to 2016/17 [4]. In that study, we assessed VE estimates by different intervals between vaccination and hospitalisation and estimated a VE of 96% (95% CI: 74%–100%) against influenza A(H1N1)pdm09 for children vaccinated no more than 2 months before hospitalisation. In the present study, we found that most children were vaccinated 14 days–2 months before hospitalisation and that the VE estimates during the early part of the season were also high. This suggests the influenza vaccine confers strong protection soon after its administration. However, in the previous study the influenza VE declined as the season progressed, emphasising the importance of appropriate timing of vaccination [11,12]. Our findings were also consistent with the recently reported VE estimate of 91% (67%-98%) against A(H1N1)pdm09 in children 1-8 years of age in Canada for 2018/19 [13].

This study had several limitations. First, we did not have data on the genetic or antigenic characteristics for the circulating influenza A(H1N1)pdm09 strains in Hong Kong to compare with the A/Michigan/45/2015(H1N1) strain included in the 2018/19 northern hemisphere formulation of the influenza vaccine. This strain was previous included in the 2017 and 2018 southern hemisphere vaccines, and the 2017/18 northern hemisphere vaccine. Nevertheless, our high VE estimates suggest that substantial antigenic drift away from the vaccine strain was unlikely. Moreover, this study may have suffered sparse data bias [14] with few vaccinated and influenza-positive cases. Finally, our estimate of high VE is largely based on protection against influenza A(H1N1)pdm09 and we were unable to estimate VE against influenza A(H3N2) or B which could affect end-of-season VE estimates if influenza A(H3N2) predominates later in the season.

## Conclusions

In early part of the 2018/19 winter influenza season in Hong Kong, we found evidence that influenza vaccination provided very good protection against laboratory-confirmed influenza A(H1N1)pdm09 virus hospitalisation in children aged 6 months–17 years.
